# Dietary supplementation with bovine-derived milk fat globule membrane lipids promotes neuromuscular development in growing rats

**DOI:** 10.1186/s12986-017-0161-y

**Published:** 2017-01-23

**Authors:** James F. Markworth, Brenan Durainayagam, Vandre C. Figueiredo, Karen Liu, Jian Guan, Alastair K. H. MacGibbon, Bertram Y. Fong, Aaron C. Fanning, Angela Rowan, Paul McJarrow, David Cameron-Smith

**Affiliations:** 1 Liggins Institute, The University of Auckland, Private Bag 92019, Auckland, 1142 New Zealand; 2 Fonterra Research and Development Centre, Private Bag 11029, Palmerston North, 4442 New Zealand; 3 Fonterra Co-operative Group Ltd, Palmerston North, 4442 New Zealand

**Keywords:** Muscle, Nerve, Myogenesis, Complex milk lipids, Agrin

## Abstract

**Background:**

The milk fat globule membrane (MFGM) is primarily composed of polar phospho- and sphingolipids, which have established biological effects on neuroplasticity. The present study aimed to investigate the effect of dietary MFGM supplementation on the neuromuscular system during post-natal development.

**Methods:**

Growing rats received dietary supplementation with bovine-derived MFGM mixtures consisting of complex milk lipids (CML), beta serum concentrate (BSC) or a complex milk lipid concentrate (CMLc) (which lacks MFGM proteins) from post-natal day 10 to day 70.

**Results:**

Supplementation with MFGM mixtures enriched in polar lipids (BSC and CMLc, but not CML) increased the plasma phosphatidylcholine (PC) concentration, with no effect on plasma phosphatidylinositol (PI), phosphatidylethanolamine (PE), phosphatidylserine (PS) or sphingomyelin (SM). In contrast, muscle PC was reduced in rats receiving supplementation with both BSC and CMLc, whereas muscle PI, PE, PS and SM remained unchanged. Rats receiving BSC and CMLc (but not CML) displayed a slow-to-fast muscle fibre type profile shift (MyHCI → MyHCIIa) that was associated with elevated expression of genes involved in myogenic differentiation (myogenic regulatory factors) and relatively fast fibre type specialisation (*Myh2* and *Nfatc4*). Expression of neuromuscular development genes, including nerve cell markers, components of the synaptogenic agrin–LRP4 pathway and acetylcholine receptor subunits, was also increased in muscle of rats supplemented with BSC and CMLc (but not CML).

**Conclusions:**

These findings demonstrate that dietary supplementation with bovine-derived MFGM mixtures enriched in polar lipids can promote neuromuscular development during post-natal growth in rats, leading to shifts in adult muscle phenotype.

**Electronic supplementary material:**

The online version of this article (doi:10.1186/s12986-017-0161-y) contains supplementary material, which is available to authorized users.

## Background

Skeletal muscle tissue comprises a spectrum of muscle fibre types that differ in structure, molecular composition and functional properties [1, 2]. The fibre type profile of a given muscle is initially established during embryonic development, independently of neural influences, by the differentiation of distinct myoblast lineages, which are committed to form different myofibre type populations [2, 3]. However, nerve activity subsequently plays a major role in the maintenance and modulation of the muscle fibre type profile during early post-natal development and throughout adult life [4–6]. Muscle fibre characteristics may be altered, changing size and transitioning from a fast-to-slow or a slow-to-fast fibre type profile both during normal post-natal development and in the adult organism in response to exercise, disuse and ageing [6–9]. Nutrition is an additional known, but less well understood, determinant of muscle fibre type profile [8].

Early post-natal life is a period of heightened neuromuscular plasticity, in which marked morphological changes occur in pre- and post-synaptic components of the neuromuscular junction (NMJ) [10]. These changes coincide with shifts in muscle fibre type profile as specific muscles develop their adult phenotype [2]. During embryonic development, acetylcholine (ACh) receptors (AChRs) accumulate in a broad band in the middle portion of the myofibre and extensive innervation occurs, generating polyinnervated NMJs with large motor units [11]. Subsequent maturation of the NMJ during post-natal development involves extensive reorganisation of pre- and post-synaptic morphology and a removal of surplus axonal terminals at polyinnervated NMJs. The compound agrin, released from pre-synaptic axon nerve terminals, plays a key role in NMJ maturation during post-natal development by stimulating clustering and aggregation of AChRs on the muscle fibre membrane. Multiple alternatively spliced forms of agrin that differ in their binding characteristics and bioactivity are synthesized by both nerve and muscle cells [12]. Neurons specifically express splice variants of agrin (Z^+^ isoforms) which include sequences encoded by the alternate exons 32 and/or 33 [12, 13]). Notably, these Z+ isoforms are 1,000-fold more active than those lacking Z exons (Z^−^ isoforms) in inducing aggregation of AChR clusters at the NMJ [14]. Synaptic agrin is cleaved and inactivated by neurotrypsin, resulting in the release of C-terminal agrin fragments (CAFs). Plasma CAFs have been found to be elevated in a subset of human sarcopenia patients, suggesting that uncontrolled degradation of agrin at the NMJ may contribute to age-associated neuromuscular decline [15–18]. Similarly, overexpression of neurotrypsin resulted in a precocious sarcopenic phenotype that was characterised by weakness, NMJ fragmentation and pathological muscle abnormalities (termed SARCO mice) [19]. Administration of a recombinant neurotrypsin-resistant form of agrin has been shown to improve muscle pathology by minimising the disassembly of the NMJ in SARCO mice [20]. Therefore, modulation of muscle agrin signalling and/or NMJs may be a novel treatment of sarcopenia and neuromuscular disease.

Milk is the main source of nutrition in newborn mammals. It also contains a number of minor constituents that possess potential bioactivity beyond their pure nutritional significance [21, 22]. Milk fat is secreted as lipid droplets consisting of a hydrophobic triglyceride core enclosed within a thin membrane that is composed of a lipid and protein trilayer and is known as the milk fat/lipid globule membrane (MFGM) [22]. The MFGM contains polar lipids including phospholipids (PLs) [phosphatidylserine (PS), phosphatidylcholine (PC), phosphatidylethanolamine (PE), phosphatidylinositol (PI)], sphingolipids [sphingomyelin (SM)] and gangliosides (GM3 and GD3) [21–23]. Although PLs and sphingolipids represent only 0.5–1% of the total lipids in milk, there is considerable interest in these compounds due to their potential bioactivity and because several health-promoting effects have been attributed to MFGM composites [21, 22, 24, 25]. In particular, MFGM composites including PLs [24], sphingolipids [26, 27] and gangliosides [28–30] have purported positive effects on neuroplasticity. For example, dietary supplementation with bovine-derived MFGM mixtures has recently been reported to promote post-natal neurodevelopment [31–35] and to limit age-associated neural decline in elderly animals [36].

Given the close relationship between nerve and muscle, MFGM supplementation may have potential to indirectly influence muscle tissue, secondary to effects on neuroplasticity. Additionally, components enriched in the MFGM, including PLs and sphingolipids, have recently been implicated as molecules that play an important direct positive role in muscle cell growth and development [37–39]. In a recent study, combined habitual exercise and dietary supplementation with a bovine-derived MFGM supplement containing both MFGM lipids and MFGM proteins was reported to minimise age-associated loss of muscle mass and force-generating capacity in senescence-accelerated mice (SAMP1 mice) [40]. Furthermore, several recent human clinical trials have reported apparent potential beneficial effects of dietary MFGM supplementation on neuromuscular function when combined with exercise training [41–43]. However, whether dietary supplementation with MFGM mixtures has direct effects on skeletal muscle plasticity, the specific MFGM components that exert bioactivity and the underlying mechanisms involved remains unclear.

In the present study, we examined the effect of dietary supplementation with bovine-derived MFGM mixtures, differing in the composition of PLs/sphingolipids and presence of MFGM proteins, during post-natal development on neuromuscular plasticity in growing rats. Specifically, we analysed the effect of feeding MFGM during development on the resulting adult muscle phenotype and the expression of the myogenic, synaptogenic and fibre type specialisation genes that are central to neuromuscular development.

## Methods

### Animal experiments

Experiments were approved by the Animal Ethics Committee, The University of Auckland (001260). Eight independent litters of Wistar rats were received from the animal resource unit of the University of Auckland at post-natal (PN) day 7. The litter size was normalised at birth to eight male pups per litter. At PN day 10, the pups were randomly allocated to receive dietary supplementation with blank gelatin (BG) or gelatin cubes formulated with one of three different bovine-derived MFGM mixtures (details below) (supplied by Fonterra Co-operative Group Limited). The pups were weaned at PN day 21 and experiments were terminated at PN day 70.

### MFGM formulation and dietary supplementation

Gelatin cubes were used for administration of the dietary supplements, which were formulated using raspberry flavouring and gelatin (10% w/v) containing sucrose (10% w/v) as previously described [32, 33, 36, 44]. Briefly, the MFGM mixtures were dissolved in water using a food processor and were then mixed with 1 L of gelatin/sucrose mixture at 50 °C. The mixture was then transferred into ice cube trays and was firmed at 4 °C. Each cube contained 12.5 mL of gelatin/sucrose solution, with or without an added MFGM mixture. Three different MFGM mixtures were tested; they were administered at various absolute doses so as to match the administered amount of gangliosides (GD3), an established brain bioactive molecule [35]. The bovine-derived MFGM supplements tested included a complex milk lipid (CML) mixture (reported previously in [33, 35, 44]), beta serum concentrate (BSC) (reported previously in 32) and a polar-lipid-enriched complex milk lipid concentrate (CMLc, “PGC80”) that lacks an MFGM protein component (reported previously in 36). The dose of CML used and the standardised ganglioside concentration were based on our previously published study in which developing rats received dietary supplementation with CML containing 5.9% gangliosides, at a dose of 1% w/w of dietary intake [35]. The gross compositions of the three different MFGM mixtures are described in Table 1.

**Table 1:** Compositions of the supplements (g/100 g)

	CML	BSC	CMLc
Protein	7.0	52.3	0.0
MFGM protein present	N	Y	N
MFGM structure present	N	Y	N
Total fat	80.0	36.2	88.0
PLs	45.2	13.7	71.7
PC (% total PLs)	14.5	27.0	23.1
PE (% total PLs)	27.2	29.2	34.7
PI (% total PLs)	18.7	8.8	8.8
PS (% total PLs)	26.1	12.1	12.0
Ganglioside GD3	4.8	0.63	1.8
PL:GD3 ratio	9.4	21.7	39.8
Lactose	3.0	6.6	3.2
Minerals	8.0	5.2	9.1
PLs (mg/g/day)	=0.64 × 45.2/100 (0.29)	=5.05 × 13.7/100 (0.69)	=1.78 × 71.7/100 (1.28)

*CML* complex milk lipids, *BSC* beta serum concentrate, *CMLc* complex milk lipid concentrate, *MFGM* milk fat globule membrane, *PL* phospholipid, *PC* phosphatidylcholine, *PE* phosphatidylethanolamine, *PI* phosphatidylinositol, *PS* phosphatidylserine, *SM* sphingomyelin

A total of 64 animals (eight pups from eight independent litters) were studied (*n* = 16 per group). Treatment was performed within litter, to minimise the effect of between-litter variability. On PN day 10, two pups within each litter of eight were randomly allocated into one of the four treatment groups. The rats were hand fed from PN day 10 until PN day 21 (weaning day) and were individually cage fed thereafter until PN day 69 (week 10). The dose of MFGM supplementation was calculated daily based on animal body weight. The doses of CML, BSC and CMLc were 0.64, 5.05 and 1.78 mg/g/day respectively. The control group was fed with BG cubes, which were prepared identically except without the addition of any MFGM mixture. The rats were single caged with the allocated gel treatment for 1–2 h each day and were returned to their home cage after they had consumed the gels.

### Tissue collections

On PN day 70, the rats were deeply anaesthetised with pentobarbital (125 mg/kg, i.p.) and blood samples (1–2 mL) were collected via cardiac punctures using heparin as an anticoagulant. Blood plasma was separated by centrifugation prior to storage at −80 °C. The rats were transcardially perfused with normal saline until the outflow from the heart ran clear. The posterior musculature of the lower left hind limb (*triceps surae*) was dissected and the soleus muscle was removed intact. The soleus muscle was weighed and then cut in half transversely at the mid belly. One portion of the soleus was snap frozen in liquid nitrogen and stored at −80 °C for molecular analysis. The remaining half was covered in Cryoglue optimum cutting temperature (OCT) compound (SLEE medical, Mainz, Germany) and rapidly frozen in dry-ice-cooled isopentane for subsequent immunohistological analysis.

### Immunohistochemical analysis of muscle tissue

Cross-sections (10 μm) were cut from the mid belly of OCT-embedded soleus muscles in a cryostat at −20 °C (Leica CM3050 S, Leica Biosystems, Nussloch, Germany). Tissue sections were adhered to Superfrost^TM^ Plus slides (Thermo Fisher Scientific, Waltham, MA, USA) and were air-dried at room temperature. The slides were blocked in 10% goat serum (GS; Vector Laboratories, Burlingame, CA, USA) in phosphate buffered saline (PBS) at room temperature for 2 h, prior to overnight incubation with a cocktail of immunoglobulin (Ig) sub-class specific mouse monoclonal primary antibodies against MyHCI (BA-F8, mouse IgG2b, diluted 1:12.5), MyHCIIa (SC-71, mouse IgG1, 1:600) and MyHCIIb (BF-F3, mouse IgM, 1:25) as previously described [45]. An antibody against laminin (2E8, mouse IgG2a, 1:50) was used simultaneously to stain the basal lamina surrounding each myofibre. Monoclonal antibodies were obtained from the Developmental Studies Hybridoma Bank, created by the NICHD of the NIH and maintained at The University of Iowa, Department of Biology, Iowa City, IA, USA. This method allows for visualisation of all four rodent MyHC isoforms simultaneously, together with the muscle fibre boundaries, on a single tissue section because MyHCIIx fibres remained unstained [45]. All primary antibodies were prepared in 10% GS solution in PBS. Following overnight primary antibody incubation, the slides were washed five times, for 5 min each time, in PBS prior to incubation with Alexa Fluor goat anti-mouse secondary antibodies (Alexa Fluor 350 IgG2b, Alexa Fluor 488 IgG1, Alexa Fluor 555 IgM and Alexa Fluor 647 IgG2a; Life Technologies, Carlsbad, CA, USA) for 2 h at room temperature. All secondary antibodies were diluted 1:500 in PBS. Following five further washes, for 5 min each time, in PBS, the slides were mounted with coverslips in Immu-Mount aqueous mounting medium (Thermo Fisher Scientific, Waltham, MA, USA). Antibody staining was visualised and images were captured at 10× magnification using an upright fluorescence microscope (Axio Imager Z2, Carl Zeiss, Oberkochen, Germany) equipped with a fully motorised automatic stage (VSlide Scanner, MetaSystems, Alltlussheim, Germany). Sequential images, spanning the entire area of the muscle cross-section, were captured and were then tiled into a single composite image using V-slide software. Global linear adjustments to the image fluorescent signal brightness and contrast were made in MetaViewer software (MetaSystems Alltlussheim, Germany). Semi-automated quantitative analysis was performed using Image J software to determine the muscle fibre type and the cross-sectional area (CSA) of individual myofibres. All fibres within the muscle section were analysed. Whilst the IHC staining method used potentially allows for the identification of hybrid myofibres the image analysis procedure used includes all fibres which express MyHCIIa myosin (including hybrid MyHCI/MyHCIIa) as Type IIa fibres. Thus, whilst the quantification procedure used does not specifically allow for analysis of the specific contribution of hybrids, any hybrid fibres present would contribute to the total MyHCIIa + fibre count.

### RNA Extraction and Reverse Transcription Polymerase Chain Reaction (RT-PCR)

Total RNA was extracted from a ~ 20 mg piece of frozen soleus muscle using the RNeasy kit (Qiagen, Venlo, Netherlands) as per the manufacturer’s instructions. The RNA concentration was determined using a NanoDrop 1000 spectrophotometer. The RNA samples were diluted in nuclease-free water and first-strand cDNA synthesised from 0.5 μg of total RNA using the High-Capacity cDNA Reverse Transcription Kit (Life Technologies, Carlsbad, CA, USA). RT-PCR was performed on a LightCycler 480 using LightCycler 480 SYBR Green I Master mix (Roche Applied Science, Indianapolis, IN, USA). The RT-PCR fluorescent emission data were analysed for the cycle threshold (Ct) value. *Gapdh* was selected as an appropriate housekeeping gene and was used for normalisation and quantification of the target mRNA PCR data by the 2(−∆Ct) method. A standard curve and a melting curve were performed for each primer pair to confirm efficiency and single product amplification. Primer sequences used are presented in Additional file 1: Table S1.

### HPLC–MS Analysis of Plasma and Tissue PLs

Rat muscle samples (~50 mg) were homogenised in 1 mL of Milli Q water using 2.8 mm ceramic beads in a OMNI Bead Ruptor Homogeniser (Omni International, Kennesaw, GA, USA) (5.65 m/s, 2 × 1 min). Lipids were extracted from the rat muscle homogenate and plasma samples (0.25 mL) using the modified Svennerholm and Fredman [46] extraction protocol as described by Norris et al. [47]. The final non-polar chloroform fraction containing the PLs was made to 5 mL in choloroform/methanol (2:1) and used for PL analysis. The analysis of PLs was performed on an ACQUITY UPLC system (Waters, Milford, MA, USA), equipped with an APS-2 Hypersil column (150 mm × 2.1 mm, 3 μm, Thermo Electron Corporation, Waltham, MA, USA), and interfaced to a TSQ Quantum mass spectrometer (Thermo Electron Corporation, Waltham, MA, USA) using a heated electrospray ionisation source as previously described [44]. The PLs were detected using precursor ion or neutral losses that occur to the PLs during fragmentation [48].

### Data analysis

The data were assessed for normality using the Shapiro–Wilk test. Variables that were found not to be normally distributed were transformed (log or square root) in order to achieve the assumption of normality. Data between the four supplementation groups were compared using a one-way analysis of variance (ANOVA). Following a significant main ANOVA effect, Holm-Šidák post-hoc tests were performed to compare each of the experimental groups with the control (BG) group. The PL compositions of plasma and muscle tissue were analysed by two-way ANOVA with supplementation group and PL species class as factors. The data are presented as means ± SEM. *p <* 0.05 was considered to be statistically significant.

## Results

### Body and muscle weights

There was no significant effect of MFGM mixture supplementation group on animal body weight, soleus muscle weight or soleus weight when normalised to body weight (mg/g body weight).

### Plasma and muscle PL composition

**Fig. 1:**
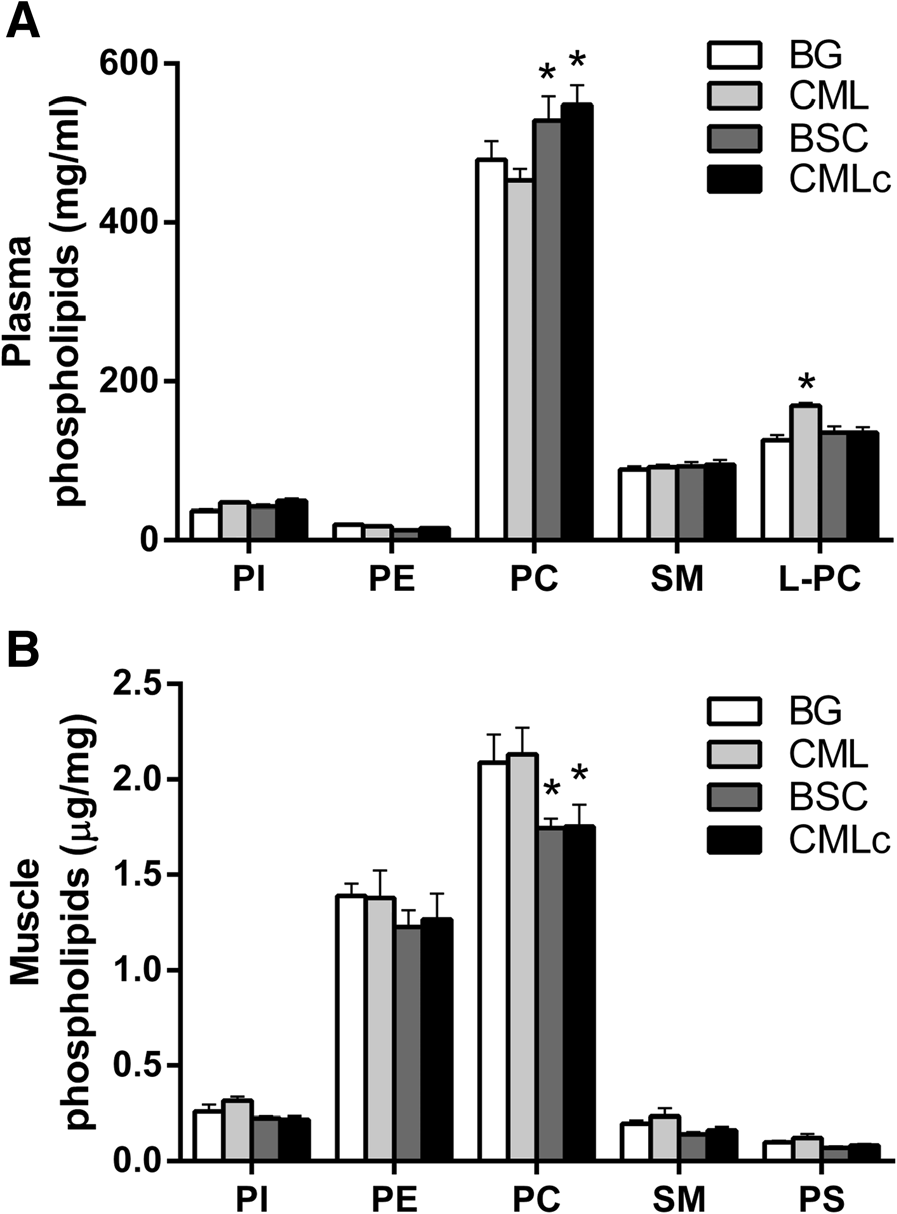
Muscle and plasma phospholipid (PL) concentrations in rats receiving dietary supplementation with various milk fat globule membrane (MFGM) mixtures. **a** Plasma concentration (mg/mL) of PL species. **b** Muscle concentration (μg/mg wet weight) of PL species

The plasma PL concentration showed a significant interaction effect (*p* < 0.001) between the MFGM supplementation group and the type of PL species (Fig. 1a). PC was by far the most abundant PL species detected in plasma and was present at significantly higher concentrations compared with the other PL species (Fig. 1a). The plasma PC concentration was futher increased above that in the BG group in both the BSC group (*p* < 0.01) and the CMLc group (*p* < 0.001) but not the CML group (Fig. 1a). The plasma concentrations of other PLs, including PI, PE and SM, were not significantly influenced by the MFGM supplementation group (Fig. 1a). However, plasma lyso-phosphatidylcholine (L-PC) was found to be elevated in the CML group (*p* < 0.05) but not in the BSC and CMLc groups (Fig. 1a). Analysis of the PL composition of skeletal muscle tissue samples showed main effects of supplementation group (*p* < 0.01) and PL species (*p* < 0.001) (Fig. 1b). Similar to plasma, PC was the predominant PL species that was detected in muscle, although, unlike in plasma, PE was also found to be a major component within the muscle tissue homgenates (Fig. 1b). Compared with the BG group, the intramuscular concentration (μg/mg) of PC was significantly lower in the BSC group (*p* < 0.01) and the CMLc group (*p* < 0.01) (Fig. 1b). In contrast, no effect of CML supplementation on the muscle PC concentration was apparent. The muscle concentrations of PI, PE, PS and SM were not influenced by dietary supplementation with any of the three MFGM mixtures (Fig. 1b).

### Muscle fibre type composition

**Fig. 2:**
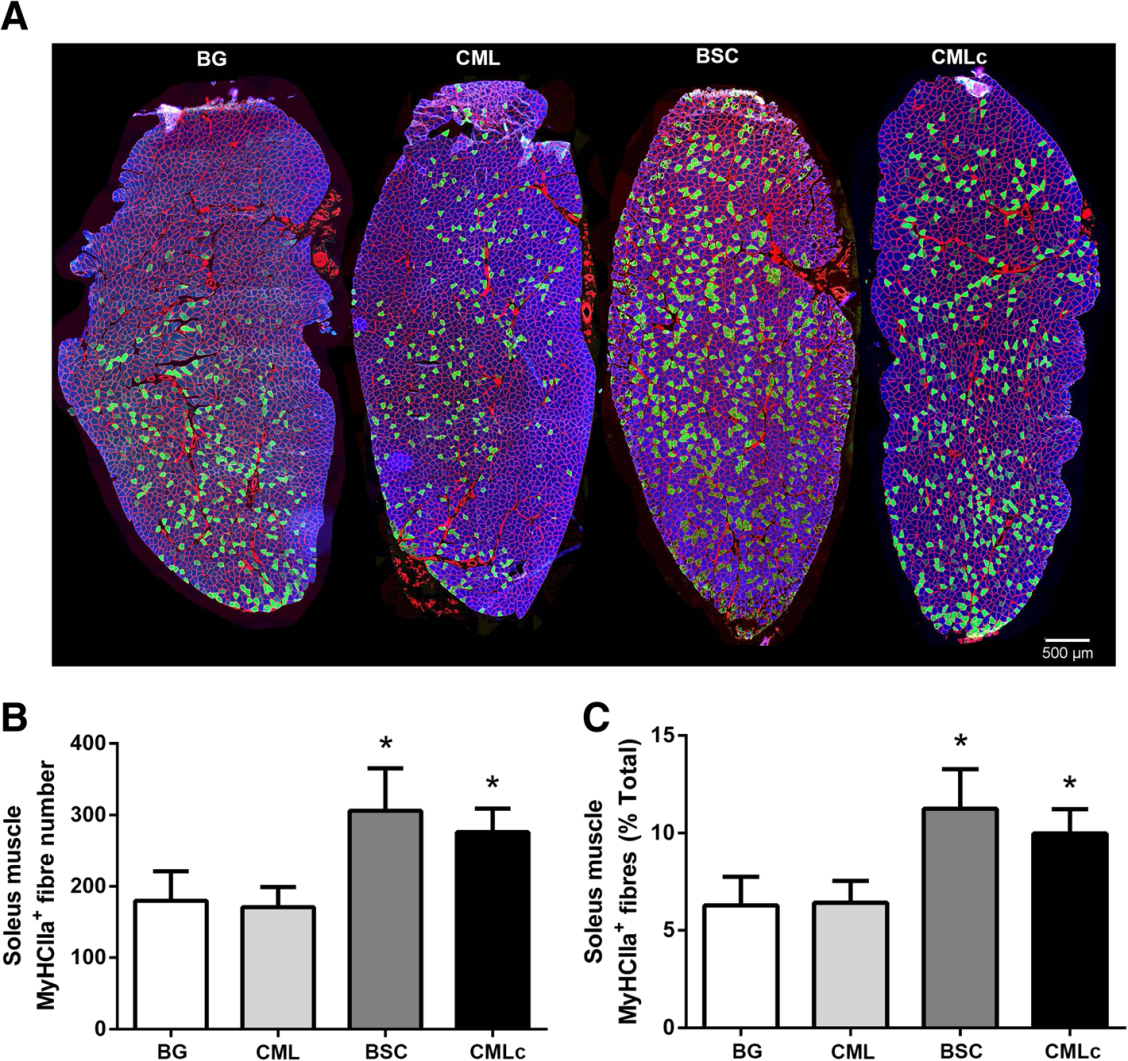
Muscle fibre type composition of the soleus muscle of rats supplemented with various milk fat globule membrane (MFGM) mixtures during post-natal development. **a** Representative images of soleus muscle cross-sections probed with mouse monoclonal antibodies against MyHCI (IgG2b, Alexa Fluor 350, *blue*), MyHCIIa (IgG1, Alexa Fluor 488, *green*), MyHCIIb (IgM, Alexa Fluor 555, *red*) and laminin (IgG2a, Alexa Fluor 647, far-*red*, pseudo coloured *red*). **b** Total number of soleus muscle fibres staining positive for MyHCIIa. **c** Percentage composition of total myofibres staining positive for MyHCIIa. * *p* < 0.05 vs BG group

There was a significant effect of supplementation group on the absolute number of MyHCIIa-positive myofibres within the soleus muscle (*p* < 0.05) (Fig. 2b). Similarly, there was a significant effect of supplementation group on the MyHCIIa fibre number expressed as the percentage of total fibres analysed (% composition of MyHCIIa-positive fibres) (*p* < 0.05) (Fig. 2c). Post-hoc analysis revealed an increase in both the absolute number (Fig. 2b) and the % composition (Fig. 2c) of MyHCIIa fibres in rats supplemented with the BSC and CMLc MFGM mixtures. In contrast, there was no significant effect of the CML MFGM mixture on the muscle fibre type composition. These data indicate a “slow-to-fast” fibre type shift from Type I (MyHCI-positive) towards Type IIa (MyHCIIa-positive) muscle fibres in the soleus muscle of rats receiving supplementation with the BSC and CMLc MFGM mixtures but not the CML MFGM mixture.

### Muscle fibre size

**Fig. 3:**
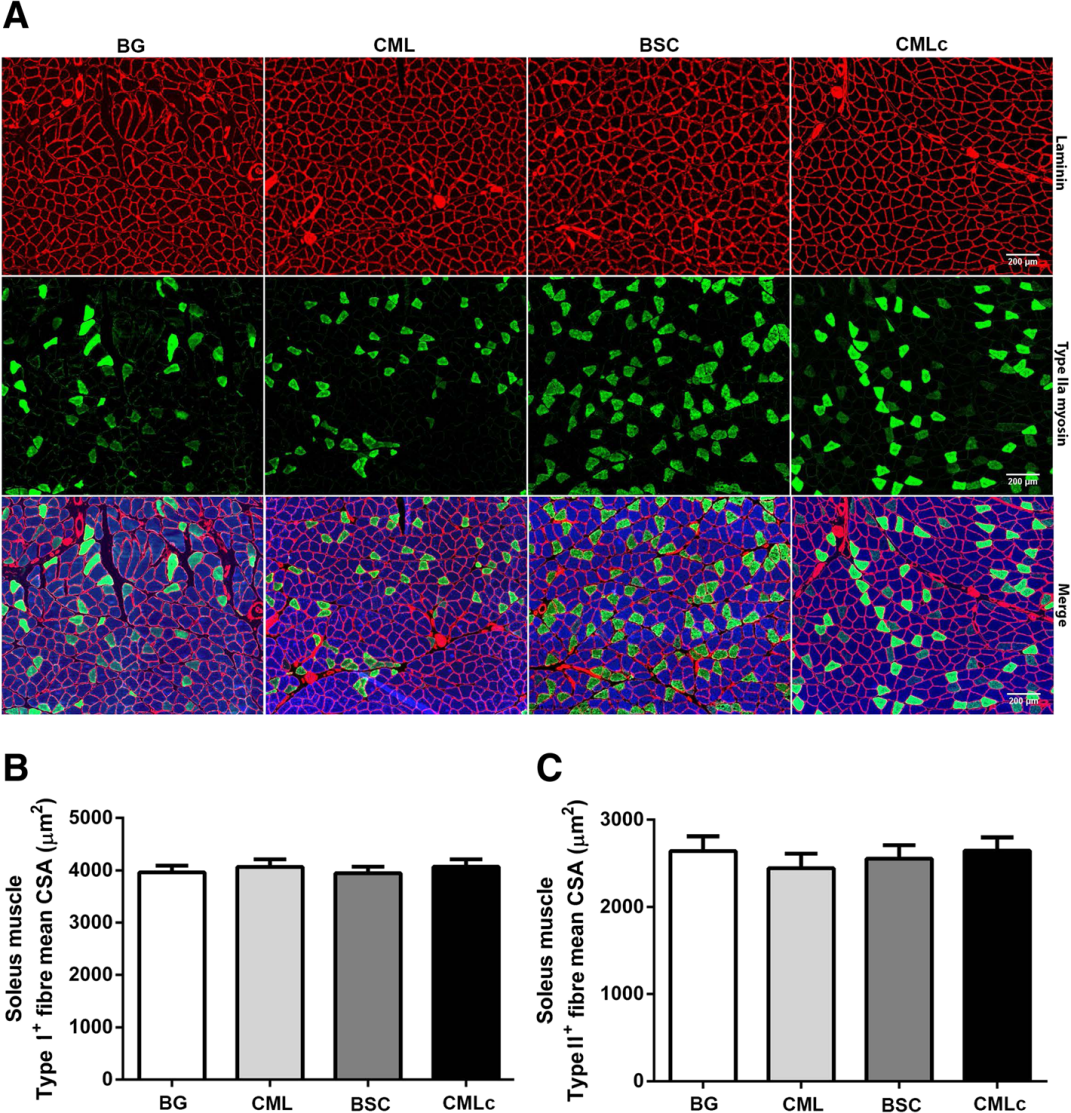
Soleus muscle myofibre cross-sectional area (CSA) (μm^2^) in rats supplemented with various milk fat globule membrane (MFGM) mixtures. **a** Representative images of soleus muscle cross-sections probed with mouse monoclonal antibodies against MyHCI (IgG2b, Alexa Fluor 350, *blue*), MyHCIIa (IgG1, Alexa Fluor 488, *green*), MyHCIIb (IgM, Alexa Fluor 555, *red*) and laminin (IgG2a, Alexa Fluor 647, far-*red*, pseudo coloured *red*). **b** Mean CSA (μm^2^) of soleus muscle Type I (MyHCI-positive) myofibres. **c** Mean CSA (μm^2^) of soleus muscle Type IIa (MyHCIIa-positive) myofibres

There was no significant effect of supplementation with any of the three MFGM mixtures on the CSA of Type I (MyHCI-positive) (Fig. 3b) or Type IIA (MyHCIIa-positive) myofibres (Fig. 3c). These data suggest that supplementation with MFGM components did not alter the soleus muscle fibre size.

### Skeletal muscle mrna expression

#### Contractile protein genes

**Fig. 4:**
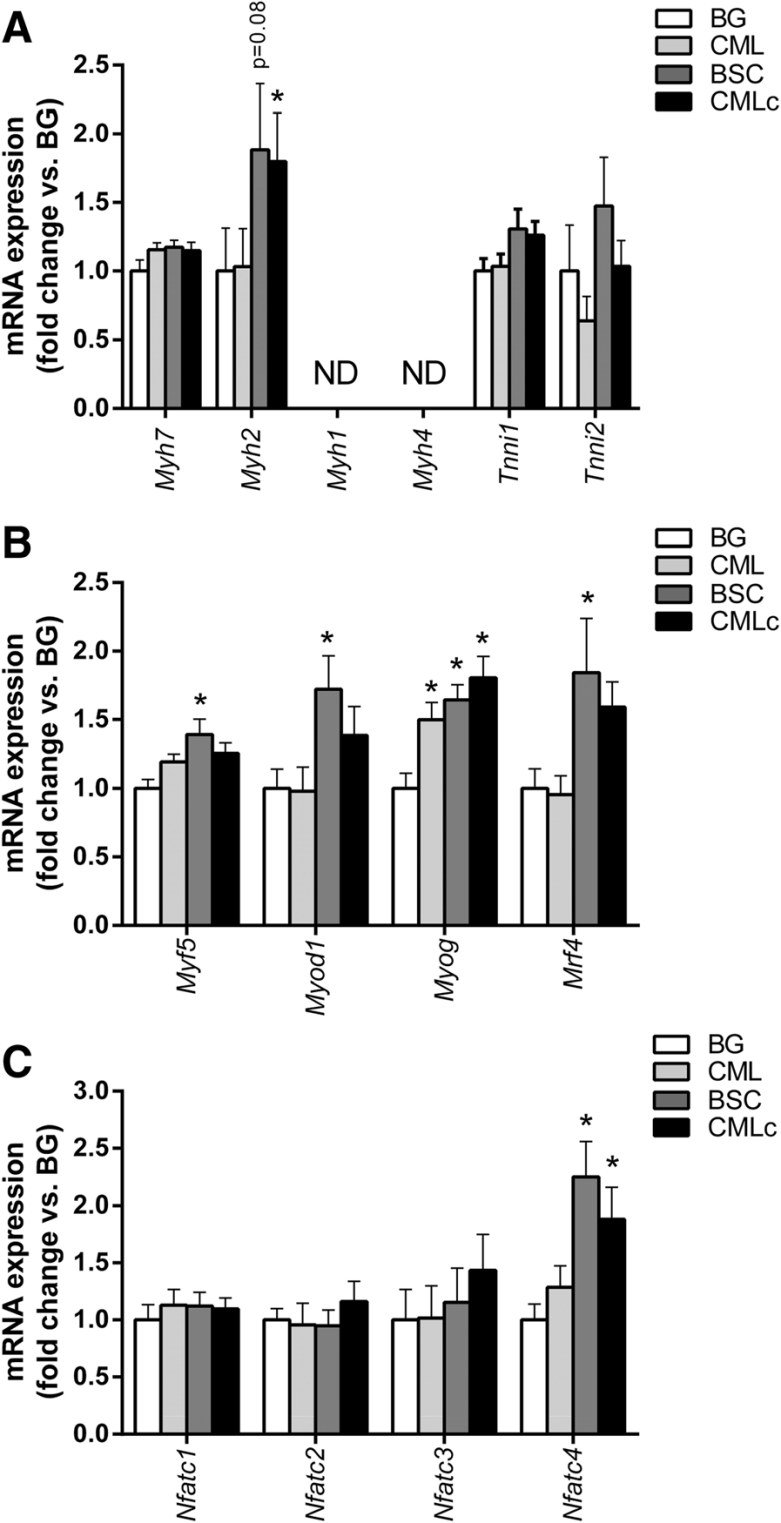
Dietary supplementation with lipid-enriched milk fat globule membrane (MFGM) mixtures influences muscle gene expression. **a** mRNA expression of transcripts encoding muscle contractile proteins in the soleus of rats receiving dietary supplementation with blank gel (BG), complex milk lipids (CML), beta serum concentrate (BSC) or complex milk lipd concentrate (CMLc). **b** mRNA expression of myogenic regulatory factor (MRF) genes in the soleus muscle of rats receiving dietary supplementation with BG, CML, BSC or CMLc. **c** mRNA expression of NFAT isoforms in the soleus muscle of rats receiving dietary supplementation with BG, CML, BSC or CMLc. Values are mean ± SEM. * *p* < 0.05 vs BG group

As expected, mRNA expression of genes encoding the slow-twitch MyHCI (*Myh7*) and intermediate-fast MyHCIIa (*Myh2*) isoforms was abundant in the predominantly tetanic slow-twitch soleus muscle. Expression of mRNA encoding the fast-twitch rodent myosin isoforms MyHCIIx (*Myh1*) and MyHCIIb (*Myh4*) was low and highly variable in this muscle (Fig. 4a). There were significant between-group differences in mRNA expression of *Myh2* in the soleus by one-way ANOVA (*p* < 0.05). Post-hoc analysis showed that *Myh2* expression was increased in the CMLc group compared with the BG group (*p* < 0.05). A similar trend towards elevated expression of *Myh2* was observed for the BSC group, but this failed to achieve statistical significance (*p* = 0.08 vs BG). Supplementation with the CML MFGM mixture had no effect on *Myh2* mRNA expression. There was no effect of any of the three MFGM mixtures on mRNA expression of the slow-twitch MyHCI isoform (*Myh7*), nor on the expression of troponin I type 1 (skeletal, slow) (*Tnni*) or troponin I type 2 (skeletal, fast) (*Tnni2*) (Fig. 4a).

#### Myogenic regulatory factors

To further explore the mechanisms by which MFGM supplementation may influence the skeletal muscle phenotype, we measured the expression of the myogenic regulatory factor (MRF) genes that are involved in skeletal muscle development and satellite cell myogenesis in mature muscle tissue (Fig. 4b). There were significant between-group differences by one-way ANOVA for muscle mRNA expression of all four MRF family members including *Myf5* (*p* < 0.05), *Myod1* (*p* < 0.05), *Myog* (*p* < 0.001) and *Myf6* (*p* < 0.05) (Fig. 4b). Post-hoc tests revealed that *Myog* mRNA levels were higher in the CML (*p* < 0.001), BSC (*p* < 0.001) and CMLc (*p* < 0.001) groups compared with the BG group. However, expression of *Myf5* (p < 0.01), *Myod1* (*p* < 0.05) and *Myf6* (*p* = 0.05) was significantly increased only in the BSC group. Similar trends towards elevated expression of *Myf5* (*p* = 0.06 vs BG) and *Myf6* (*p* = 0.16 vs BG) were observed in the CMLc group, but these failed to reach statistical significance.

#### Fibre type specialisation genes

Compared with the BG group, mRNA expression of *Nfatc4* was elevated in both the BSC group (*p* < 0.001) and the CMLc group (*p* < 0.01) (but not the CML group) (Fig. 4c). No difference between groups was observed for mRNA expression of the other NFAT isoforms, including *Nfatc1*, *Nfatc2* and *Nfatc3*.

### Neuromuscular gene expression

#### Synaptogenic genes

**Fig. 5:**
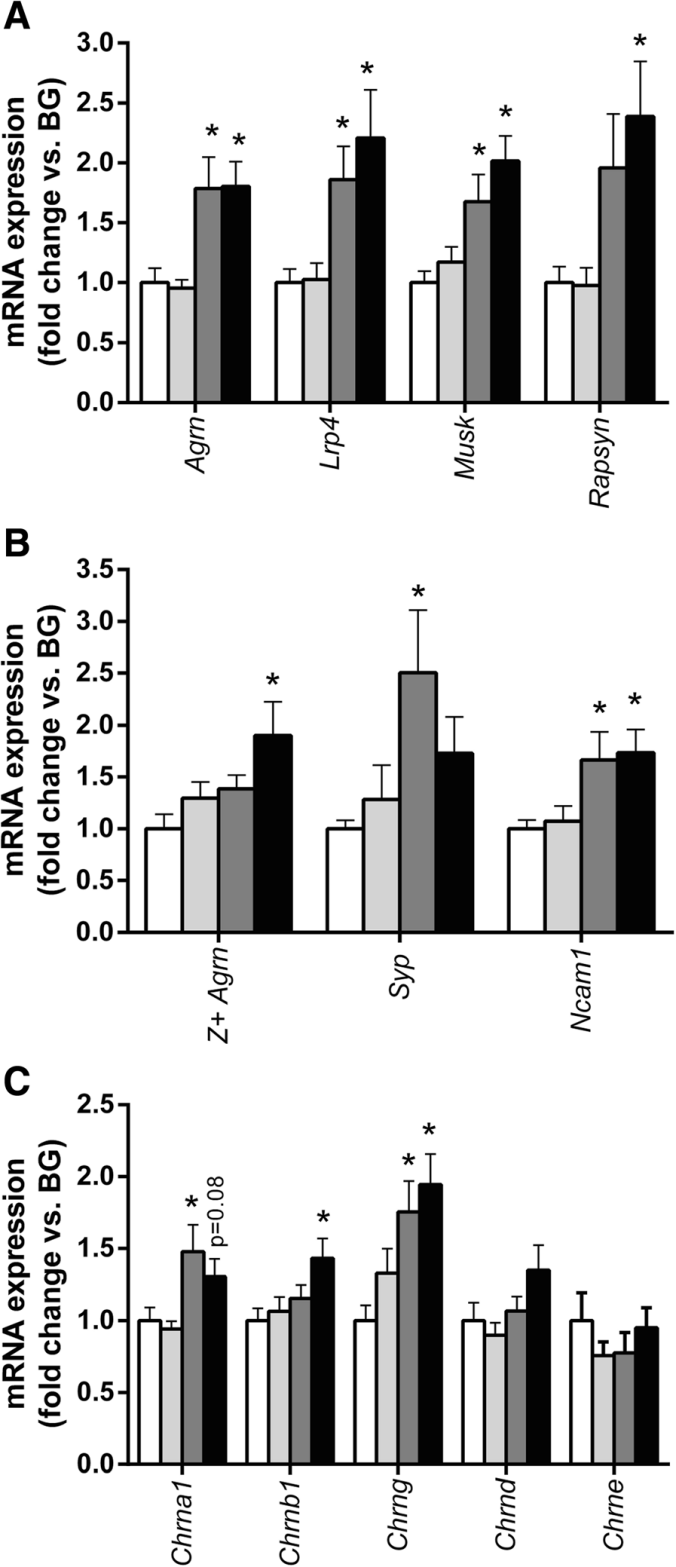
Milk fat globule membrane (MFGM) supplementation influences neuromuscular gene expression. **a** mRNA expression of components of the agrin–LRP4 pathway in the soleus muscle of rats receiving dietary supplementation with BG, CML, BSC or CMLc. **b** mRNA expression of nerve cell markers in the soleus muscle of rats receiving dietary supplementation with blank gel (BG), complex milk lipids (CML), beta serum concentrate (BSC) or complex milk lipid concentrate (CMLc). **c** mRNA expression of acetylcholine receptor (AChR) subunits in the soleus muscle of rats receiving dietary supplementation with BG, CML, BSC or CMLc. Values are mean ± SEM. * *p* < 0.05 vs BG group

mRNA expression of components of the agrin–LRP4–MuSK signalling pathway, including *Agrn* (*p* < 0.001), *Lrp4* (*p* < 0.05), *Musk* (*p* < 0.001) and *Rapsn* (*p* < 0.05), were found to be significantly different between the supplementation groups by one-way ANOVA (Fig. 5A). Post-hoc analysis showed that dietary supplementation with both BSC and CMLc increased the expression of *Agrn* (both *p* < 0.01), *Lrp4* (the *Agrn* receptor) (both *p* < 0.05) and *Musk* (CMLc *p* < 0.001, BSC *p* < 0.05). However, only the CMLc group displayed elevated muscle expression of *Rapsn*. Unlike supplementation with BSC and CMLc, supplementation with the CML mixture had no significant effect on the expression of LRP4–MuSK signalling pathway components.

#### Pre-synaptic components

The *Agrn* primer pair used above (*Agrn* 1 F*:* TTCCTCAGCAACTACAAACCTG, *Agrn* 3R*:* TTCACACACAGCACCAAAGC) amplify a region of rat *Agrn* mRNA spanning exons 1 to 3 ubiquitous to all *Agrn* splice variants (both muscle Z- and nerve Z+ isoforms). In order to determine whether mRNA of the nerve derived Z+ *Agrn* isoforms mRNA (which possess substantially greater AChR cluster aggregation bioactivity) could be detected within whole skeletal muscle samples, and whether apparent differences in *Agrn* expression were exclusively attributable to modulation of Z^−^ Agrn isoforms known to be expressed by myofibres themselves, we designed primers to specifically amplify *agrin* mRNA splice variants that include exon 32 (corresponding to protein isoforms Z8 and Z19) [49]. RT-PCR reactions with a forward primer specific for exon 30 (*Agrn* 30 F: TGTCCTGGGGGCTTCTCTGG) in combination with a reverse located within exon 32 (*Agrn* 32R: CTGGGATCTCATTGGTCAGCTC) present only in the nerve derived Z + 8 (containing exon 32 but lacking exon 33) and Z + 19 (containing both exons 32 and 33) splice variants demonstrated amplification of rat muscle tissue cDNA samples not observed in RT^−^ or non-template control samples (Fig. 5b, Additional file 2: Figure S1). Agarose gel (4%) electrophoresis of the amplified RT-PCR products revealed bands corresponding to the expected sizes of 102 bp with the *Agrn* 1 F/*Agrn* 3R (pan *Agrn*) primer pair and 149 bp with *Agrn* 30 F/*Agrn* 32R (Z+ *Agrn*) primer pair. Sequencing of these bands following gel extraction/purification confirmed that the 102 bp product generated with *Agrn* 1 F/*Agrn* 3R primers aligned with a region of rat *Agrn* between exons 1–3 and the 149 bp product with *Agrn* 30 F/*Agrn* 32R aligned with region located within alternatively spliced exon 32 (CGAGCTGACCAATGAGATCCCAG) present only in the nerve derived Z+ isoforms (Additional file 2: Figure S1). Real time RT-PCR reactions with the *Agrn* 30 F/*Agrn* 32R primer pair showed that the expression of Z^+^ *Agrn* mRNA within muscle tissue significantly differed between supplementation groups by one-way ANOVA (*p* < 0.05) (Fig. 5b). Post-hoc analysis demonstrated significantly elevated muscle Z^+^ *Agrn* mRNA expression in the CMLc group when compared to the BG group. Neither CML nor BSC supplementation had any significant effect on the expression of Z+ *Agrn* (Fig. 5b).

Consistent with our observation of detectable expression of nerve derived Z+ *Agrn* mRNA in whole muscle tissue; mRNA of the synaptic vesicle protein synaptophysin (*Syp*) was also apparently detected. Agarose gel (4%) electrophoresis of the amplified product from the real time PCR reactions (*Syp* F: TTTGCTACGTGTGGCAGCTA, *Syp* R: ACACTTGGTGCAGCCTGAAG) showed a band corresponding to the expected size of 121 bp. Sequencing of the 121 bp band following gel extraction/purification confirmed that the amplified product aligned with rat *Syp* mRNA (Additional file 2: Figure S2). Real time RT-PCR reactions with the *Syp* F/*Syp* R primer pair revealed that muscle *Syp* expression was significantly different between groups by one-way ANOVA (*p* < 0.05) with post-hoc analysis showing significantly elevated *Syp* in BSC supplemented animals. Finally, soleus mRNA expression of neural cell adhesion molecule (*Ncam1*) was also significantly different between groups (*p* < 0.01). *Ncam1* was elevated in the BSC (*p* < 0.05) and CMLc (*p* < 0.05) groups compared with the BG group (Fig. 5a). In contrast, *Ncam1* expression did not differ between the CML and BG groups.

#### Post-synaptic neuromuscular components

mRNA expression of AChR subunits alpha 1 (*Chrna1*) (*p* < 0.01), beta (*Chrnb1*) (*p* < 0.05) and gamma (*Chrng*) (*p* < 0.01) was significantly different between the supplementation groups (Fig. 5c). AChR delta (*Chrnd*) also showed a similar trend towards between-group differences, but this failed to reach statistical significance (*p* = 0.09). Post-hoc analysis revealed that *Chrng* mRNA was elevated in both the BSC and CMLc groups when compared with the BG group. Expression of *Chrna1* was also elevated only in the BSC group, although the CMLc group exhibited a similar trend (*p* = 0.08 vs BG). Only the CMLc group exhibited significantly increased muscle mRNA expression of *Chrnb1*. Expression of the AChR epsilon subunit (*Chrne*) did not differ between the MFGM supplementation groups.

## Discussion

The current study assessed the effect of dietary supplementation with different bovine-derived MFGM mixtures on post-natal neuromuscular development in growing rats. None of the MFGM supplements tested here had any effect on soleus muscle mass or myofibre size. However, a slow-to-fast shift, i.e. Type I towards Type IIa muscle fibres was observed in rats supplemented with both BSC and CMLc, which occurred as a result of an increase in both the absolute number and the relative concentration of Type IIa fibres. The effect of BSC and CMLc supplementation on muscle phenotype was associated with elevated expression of genes involved in myogenesis (MRFs), fibre type specialisation (Myh2 and NFATc4) and neuromuscular plasticity/synaptogenesis (agrin–LRP4 pathway). These findings show that dietary supplementation with bovine-derived MFGM mixtures that are enriched in polar lipids promotes neuromuscular development in rats.

Skeletal muscles are still immature at birth and important changes in the muscle fibre type profile, which coincide with maturation of the neuromuscular system, take place during early post-natal development, including the disappearance of polyneuronal innervation and the acquisition of specific motor neuron firing patterns [2]. The development and the maintenance of the fibre type profile of tetanic slow-twitch muscles (e.g. the soleus) are particularly dependent on nerve activity. In the current study, rats that received dietary supplementation with BSC and CMLc (but not CML) displayed a greater proportion of muscle fibres staining positive for the MyHCIIa isoform within the soleus muscle at PN day 70. RT-PCR analysis of muscle gene expression suggested that this effect appeared to be primarily attributable to elevated expression of the intermediate-fast MyHCIIa isoform (*Myh2* gene), with no indication of suppressed expression of the slow-twitch MyHCI isoform (*Myh7* gene). We also measured expression of the skeletal muscle contractile protein isoforms slow troponin (*Tnni1*) and fast troponin (*Tnni2*), but found that they were not influenced by MFGM supplementation. These data suggest that dietary supplementation with specific MFGM mixtures during post-natal development leads to shifts in the adult muscle fibre type profile that are primarily attributable to upregulation of the intermediate-fast Type IIa myosin isoform. An increase in the percentage of Type IIa myofibres within the soleus muscle would potentially be expected to lead to changes in muscle function including an increased force generating potential. Although no measures of muscle function were undertaken in the current study, our soleus muscle fibre type composition results may explain the previously reported positive effect of MFGM on tetanic contractile force of the soleus muscle in mice [40]. The soleus muscle is a prototypical slow-twitch muscle consisting primarily of Type I myofibres and thus a slow-to-fast fibre type shift could be seen to disrupt the normal profile of the soleus with the potential for possible negative effects on oxidative capacity. Nevertheless, Satoshi et al. 2014 reported that mice receiving MFGM supplementation displayed increased muscular endurance capacity in association with increased soleus muscle mRNA expression of oxidative genes including peroxisome proliferator-activated receptor-γ coactivator 1α (*Pgc1α*) and *Cpt-1b* [50].

Previous studies that have investigated the effect of nutrition on skeletal muscle fibre type have utilised models of energy surplus or deficit including high-fat-diet-induced obesity and caloric restriction [8]. In response to short term feeding of rodents with a high fat diet, a number of studies have reported a fast-to-slow fibre type profile shift in skeletal muscle [51–55], associated with a concurrent increase in muscle oxidative capacity [56–58]. In contrast, a single recent study reported slow-to-fast muscle fibre type transformation in response to a more prolonged (1 year) high fat diet in mice [59]. In the present study, we observed an increase in the absolute number and the relative proportion of Type IIa myofibres in the soleus muscle of rats fed BSC and CMLc, despite very little difference in overall caloric intake and macronutrient composition of the four diets. This suggests that supplementation with specific MFGM mixtures enriched in polar lipids exerted bioactive effects on the muscle fibre type profile independent of caloric/nutritional value. MFGM contains numerous potentially bioactive molecules including polar lipids as well as specific membrane proteins [21–23]. As all three tested MFGM supplements were matched for ganglioside content in the present study (and yet CML was without effect), it appears to be unlikely that the effects observed are related to the dietary intake of gangliosides. Furthermore, BSC is a complex mixture of ingredients, including a substantial MFGM protein component, which is lacking in the more purified CMLc mixture. Despite this, overall, the effects of BSC and CMLc were very similar. Thus, overall, the effects observed here also do not appear to be primarily attributable to a bioactive MFGM protein component. Although further investigation into the bioactive composites is required, collectively, these lines of evidence suggest that the polar lipid composition of bovine MFGM is a likely soure of bioactivity.

Phospholipids and sphingolipids have established bioactive properties related to neuroplasticity as well as muscle cell growth and development [37–39]. For example, cell surface exposure of PS on myoblasts is an indispensable event for myoblast fusion [60, 61] and treatment with PS liposomes can enhance muscle fibre formation *in vitro* [62]. Conversely, ablation of the PS receptor has been found to impair muscle regeneration following injury in mice [63, 64]. SM levels also relate to the state of activation of muscle myogenic precursor cells (satellite cells) [65], with SM metabolism to the metabolite sphingosine-1-phosphate (S1P) being a crucial pathway in satellite cell activation/proliferation [66–70], myoblast migration [71] and myogenic differentiation [72–77]. Furthermore, *in vivo* treatment with S1P has been shown to protect against contraction-induced muscle fatigue [78], minimise denervation-induced muscle atrophy [79] and enhance skeletal muscle regenerative capacity following traumatic injury [80]/damaging muscle contractions (exercise) [81]. Consistent with a positive role of polar lipids in the regulation of muscle cell growth and development, in the current study, we found that dietary supplementation with PL-enriched MFGM mixtures increased muscle expression of the MRFs including *Myf5*, *Myod1*, *Myog* and *Mrf4*. The MRFs play a crucial role in embryonic myogenesis, but also play multiple distinct roles in post-natal muscle, including developmental growth, fibre type control and growth/regeneration of adult myofibres in response to injury caused by mechanical loading. For example, *Myod1* is expressed more highly in fast-twitch fibres than in slow-twitch fibres and *Myod1*-null mice show shifts in fibre type of fast muscle towards a slower phenotype [82, 83]. Conversely, overexpression of an active form of *Myod1* in muscle results in a slow-to-fast fibre type conversion [84].

Nuclear factor of activated T cell (NFAT) family members are known to play a major role in converting fast and slow motor neuron stimulation patterns into specific transcriptional programs that drive muscle fibre type specialisation [85, 86].We detected expression *Nfatc1*, *Nfatc2*, *Nfatc3* and *Nfatc4* mRNA in adult soleus muscle, but found that only *Nfatc4* was induced in response to MFGM (BSC and CMLc) supplementation. An indispensable role of *Nfatc1* in the maintenance of a slow muscle phenotype is well established [86, 87]. However, more recent work has shown that, depending on the applied nerve activity pattern, different combinations of NFAT isoform expression contributes to the transcription of muscle fibre type specific genes [85]. For example, transcription of MyHC genes in the predominantly fast-twitch rat extensor digitorum longus (EDL) muscle uses different combinations of NFAT isoforms, ranging from MyHC-slow, which uses all four NFAT isoforms, to MyHCIIb, which requires only *Nfatc4* [85]. The precise role of *Nfatc4* in fibre type specialisation of the predominantly slow-twitch soleus muscle, which generally lacks expression of the MyHCIIb isoform, is unclear. Nevertheless, the notion that *Nfatc4* is specifically involved in driving the expression MyHC isoforms associated with a relatively faster muscle phenotype [85] is consistent with the Type 1 to Type IIa soleus muscle fibre type shift, concurrent to elevated *Nfatc4* expression, that was observed in response to MFGM supplementation in the current study.

Multiple splice variants of agrin exist which vary via the inclusion or exclusion of exons 32 and/or 33 (nomenclature of ref 14). Whereas most cell types synthesize agrin, only neurons produce the alternatively spliced Z+ agrin isoforms that include the sequence encoded by exons 32 and/or 33 [12]. These Z+ exons encode a domain of 8–19 amino acids that confers up to a 1,000-fold increase in AChR clustering activity relative to Z- agrin [14]. Additionally, the Z8 (with 8 additional amino acids encoded by exon 32) and Z19 (with 19 amino acids encoded by exons 32 and 33) isoforms are 150-fold more potent in promoting AChR clustering than isoforms lacking exon 32 and 45-fold more potent than the Z11 isoform (including exon 33 alone) [88–90]. Using a primer pair spanning exons 1–3 of rat *Agrn* mRNA (which is present in all splice variants) we found that *Agrn* mRNA expression was increased in the muscle of rats receiving dietary supplementation with BSC or CMLc, but not CML. Since Z- agrin variants have previously been found to be the only isoforms expressed within skeletal muscle tissue [12], this finding likely reflects an effect of MFGM supplementation on muscle derived agrin. However, using primers designed to specifically amplify *Agrn* mRNA splice variants that include exon 32 (corresponding to protein isoforms Z8 and Z19) we were apparently also able to detect expression of the Z+ *Agrn* mRNA within the rat soleus muscle. Gel electrophoresis confirmed a band of the expected size which aligned with the region within spliced exon 32 following band extraction and sequencing. On the basis of previous reports [12], we can only presume that Z+ *Agrn* mRNA expression detected was derived from motor neuron axons and/or presynaptic motor nerve terminals located within the muscle tissue. Rats receiving CMLc supplementation appeared to display significantly increased expression of Z+ *Agrn* when compared to BG, suggesting increased Agrn expression in CMLc supplemented animals may be at least partially due to induction nerve *Agrn.* Consistent with this hypothesis was detection and modulation of mRNA expression of the pre-synaptic nerve terminal marker synaptophysin within rat muscle. Increased muscle *Agrn* and neural *Z+ Agrn* mRNA expression in the soleus muscle tissue of MFGM supplemented rats was also accompanied by elevated muscle expression of downstream components of the post-synaptic agrin–LRP4 signalling pathway, including the agrin receptor *Lrp4*, the associated receptor tyrosine kinase *Musk* and the intracellular AChR-clustering protein *Rapsn*. The role of agrin in the determination of muscle phenotype has been investigated recently in mice in which the levels of agrin at the NMJ were reduced by overexpression of neurotrypsin enzyme, which cleaves active agrin to form inactive CAFs (SARCO mice) [19]. Young adult SARCO mice exhibit a precocious sarcopenic phenotype that is characterised by loss of muscle force, destabilisation of NMJs and a preferential loss of fast-twitch type II myofibres [19]. Consistently, loss of agrin function as the result of a point mutation results in deterioration of the NMJ, leads to muscle fibre atrophy and leads to a fast-to-slow muscle fibre type shift [91]. Plasma CAFs have been found to be elevated in elderly humans, suggesting that they may be a useful biomarker of sarcopenia arising from age-associated neuromuscular decline [15–18]. Interestingly, injection of an agrin biologic (NT-1654) was able to reverse the sarcopenia-like phenotype in SARCO mice, suggesting that modulating the agrin–LRP4–MuSK pathway may be a novel therapeutic strategy in the treatment of age-related muscle wasting, neuromuscular disease and associated muscle dysfunction [20]. To our knowledge, the present study is the first report of the modulation of muscle agrin levels as a result of nutritional supplementation. These findings suggest that dietary ingestion of, or dietary supplementation with, bovine MFGM or composite material could be a potential therapeutic means of manipulating the agrin–LRP4 pathway in muscle tissue.

Previously, Haramizu et al. reported the effect of dietary supplementation with an MFGM mixture in combination with habitual exercise in age related muscle dysfunction using a model of senescence-accelerated mice (SAMP1 mice) [40]. SAMP1 mice that received combined MFGM and exercise exhibited increased muscle mass (quadriceps) and contractile force (soleus and EDL) [40]. Interestingly, microarray analysis of the quadriceps muscle revealed that transcripts related to the biological process of “nervous system development and function” were most differentially expressed between the MFGMEx group and the control SAMP1 group [40]. Subsequent verification by PCR analysis showed significant induction of the neuromuscular/synaptogenic genes *Musk* and *Dok7*, as well as the myogenic genes *Myod1* and *Myog*, in response to combined MFGM supplementation and habitual exercise [40]. However, no significant differences were found in response to MFGM supplementation when administered in the absence of habitual exercise [40]. In support of the findings of Haramizu et al. [40], we report induction of several of the same genes (*Myod1*, *Myog*, *Musk*) or closely related genes (e.g. other MRFs and agrin–LRP4 pathway components) in muscle tissue of rats supplemented with BSC and CMLc, even in the absence of an exercise stimulus. To our knowledge, the current study is the first report of a direct effect of MFGM supplementation on the expression of genes related to nervous system development independent of an exercise stimulus. As early post-natal life is a stage of neuromuscular plasticity, direct effects of dietary MFGM supplementation on skeletal muscle tissue may be more readily observed in this model. However, in adult skeletal muscle, it is possible that a remodelling stimulus (e.g. exercise) may be necessary for MFGM supplementation to exert a modulatory bioactive effect.

## Conclusion

In conclusion, we found that dietary supplementation with specific bovine-derived MFGM mixtures, including BSC and CMLc, which are enriched in polar lipid components, promotes neuromuscular development in growing rats, leading to shifts in the adult’s muscle phenotype. These findings suggest that previously reported effects of MFGM or composite molecules on neuroplasticity may extend beyond the central nervous system and may impact peripheral tissues including skeletal muscle. On the basis of these findings, ingestion of dietary sources of MFGM or supplementation with bovine-derived MFGM mixtures enriched in bioactive lipids, or isolated specific bioactive MFGM components, may exert potential positive effects on skeletal muscle structure and function in physiological settings of development, growth and muscle regeneration/remodelling (e.g. tissue injury/healing, exercise recovery, reloading following disuse). Furthermore, the apparent positive effect of MFGM supplementation on modulation of neuromuscular/synaptogenic development pathways may be indicative of a potential therapeutic benefit of bovine MFGM or composite molecules in settings of nerve-related muscle dysfunction such as ageing and neuromuscular disease.

## Additional files

Additional file 1: Table S1. Primer pairs used for real time RT-PCR analysis of rat soleus muscle mRNA expression. (PDF 109 kb)
Additional file 2: Figure S1. Real time RT‐PCR analysis of Agrn and Z+ Agrn expression in rat soleus muscle. **Figure S2.** Real time RT‐PCR analysis of synaptophysin expression in rat soleus muscle. A (PDF 369 kb)

## Abbreviations

ACh: Acetylcholine
AChR: Acetylcholine receptor
BG: Blank gel
BSC: Beta serum concentrate
CAFs: C-terminal agrin fragment
CML: Complex milk lipids
CMLc: Complex milk lipid concentrate
HPLC: High performance liquid chromatography
Ig: Immunoglobulin
IHC: Immunohistological
MFGM: Milk fat globule membrane
MRF: Myogenic regulatory factor
MS: Mass spectrometry
MyHC: Myosin heavy chain
NMJ: Neuromuscular junction
OCT: Optimal cutting temperature
PC: Phosphatidylcholine
PE: Phosphatidylethanolamine
PI: Phosphatidylinositol
PL: Phospholipid
PS: Phosphatidylserine
SAMP1 mice: Senescence –accelerated mice
SM: Sphingomyelin
