# Supplementary material for: Dietary supplementation with bovine-derived milk fat globule membrane lipids promotes neuromuscular development in growing rats
Source: Nutr Metab (Lond). 2017 Jan 23;14:9. doi: 10.1186/s12986-017-0161-y (PMC5259894; doi:10.1186/s12986-017-0161-y)
Supplement: Additional file 1: Table S1. — Primer pairs used for real time RT-PCR analysis of rat soleus muscle mRNA expression. (PDF 109 kb) [file 12986_2017_161_MOESM1_ESM.pdf]

RefSeq	Gene name	Symbol	Primer sequence	
NM_017184.1	Troponin I type 1 (skeletal, slow), mRNA	*Tnni1*	Forward	GAAGACACAGAAAAGGAGCGG
			Reverse	GAAGAGCACTCTAAGCACCTCT
NM_017185.1	Troponin I type 2 (skeletal, fast), mRNA	*Tnni2*	Forward	GGAGATGAGGAGAAGCGCAA
			Reverse	GCGATCTGGAGCATCACACT
NM_017240.2	Myosin, heavy chain 7, cardiac muscle, beta (MHCI), mRNA	*Myh7*	Forward	AACAGGCCAACACCAACCTG
			Reverse	CTACTCTTCATTCAGGCCCTTGG
NM_001135157.1	Myosin, heavy chain 2, skeletal muscle, adult (MHCIIa), mRNA	*Myh2*	Forward	GCCGCGAGGTTCACACTAAA
			Reverse	TTTGTGCCTCTCTTCGGTCA
NM_001135158.1	Myosin, heavy chain 1, skeletal muscle, adult (MHCIIx), mRNA	*Myh1*	Forward	TGCATCCCTAAAGGCAGACTC
			Reverse	TTCTGAGCCTCGATTCGCTC
NM_019325.1	Myosin, heavy chain 4, skeletal muscle, adult (MHCIIb), mRNA	*Myh4*	Forward	CATCTGGTAACACAAGAGGTGC
			Reverse	GGCTTGTTCTGAGCCTCGAT
NM_001106783.1	Myogenic factor 5 (Myf5), mRNA	*Myf5*	Forward	GCCCTGATGTATCAAACGCATGT
			Reverse	TGTCCTGAAGAGCCAACTCG
NM_176079.1	Myogenic differentiation 1 (MyoD), mRNA	*Myod1*	Forward	GATGGCATGATGGATTACAGCG
			Reverse	CCACTATGCTGGACAGGCAG
NM_017115.2	Myogenin (Myog), mRNA	*Myog*	Forward	GTCCCAACCCAGGAGATCAT
			Reverse	CCACGATGGACGTAAGGGAG
NM_013172.2	Myogenic factor 6 (Myf6), mRNA	*Myf6*	Forward	TAAGGAAGGAGGAGCAAGCG
			Reverse	GGGAGTTTGCGTTCCTCTGA
NM_175754.1	Agrin, mRNA	*Agrn*	Forward	TTCACACACAGCACCAAAGC
			Reverse	TTCCTCAGCAACTACAAACCTG
	Z+ Agrin, mRNA	*Z+ Agrn*	Forward	TGTCCTGGGGGCTTCTCTGG
			Reverse	CTGGGATCTCATTGGTCAGCTC
NM_031322.3	Low density lipoprotein receptor-related protein 4 (Lrp4), mRNA	*Lrp4*	Forward	TTCACGGATCCTGGAATGGG
			Reverse	GTCAGGTCCACCCTCTTTCTT
NM_031061.1	Muscle, skeletal, receptor tyrosine kinase, mRNA	*MusK*	Forward	AGCGTGGACATTCCAAACCT
			Reverse	GGAGAGCAAACACCGCAAAG
NM_001108584.1	Receptor-associated protein of the synapse, mRNA	*Rapsn*	Forward	CAGTTTCTACGCCCAGGTCA
			Reverse	CCTAGGGAAGGCTGTCTCCA
NM_024485.1	Cholinergic receptor, nicotinic, alpha 1 (muscle) (AChR alpha 1), mRNA	*Chrna1*	Forward	GGTGGCCATCAATCCGGAAA
			Reverse	TGGGGCAGCAGGAGTAAAAC
NM_012528.1	Cholinergic receptor, nicotinic, beta 1 (muscle) (AChR beta 1), mRNA	*Chrnb1*	Forward	CAGCCTGAACGAGAAGGATGAA
			Reverse	GTCCGGGAGCCAAACAGATT
NM_019145.1	Cholinergic receptor, nicotinic, gamma (muscle) (AChR gamma), mRNA	*Chrng*	Forward	TGGGAAACAATGTGGACGGT
			Reverse	CTGCCAATCGAAGGGGAAGT
NM_019298.1	Cholinergic receptor, nicotinic, delta (muscle) (AChR delta), mRNA	*Chrnd*	Forward	ACAACCGCAGTTACCCCATT
			Reverse	TCCCACTCACCGTTCTCTGT
NM_017194.1	Cholinergic receptor, nicotinic, epsilon (muscle) (AChR epsilon), mRNA	*Chrne*	Forward	ACCGCAGCTTTTACCGAGAA
			Reverse	CGACGGATGATGAGCGTGTA

NM_031521.1	Neural cell adhesion molecule 1, mRNA	*Ncam1*	Forward	CCAAACCATGACGGAGGGAA
			Reverse	TCTACAGGACCCTTCTCGGG
NM_012664.2	Synaptophysin, mRNA	*Syp*	Forward	TTTGCTACGTGTGGCAGCTA
			Reverse	ACACTTGGTGCAGCCTGAAG
NM_001244933.1	Nuclear factor of activated T-cells, cytoplasmic, calcineurin-dependent 1, mRNA	*Nfatc1*	Forward	AATAACCAGCCCCGTCCAAG
			Reverse	GGTCAGAGCTGGCTCAAAGT
NM_001107805.1	Nuclear factor of activated T-cells, cytoplasmic, calcineurin-dependent 2, mRNA	*Nfatc2*	Forward	CCAGACTTACCTGGATGACGTTAAT
			Reverse	TAGAAGGCGTCGTGCGATAC
NM_001108447.1	Nuclear factor of activated T-cells, cytoplasmic, calcineurin-dependent 3, mRNA	*Nfatc3*	Forward	TTGGAACACCAGCCATCAGG
			Reverse	CTCGTTCACCTCTAGATTCGCC
NM_001107264.1	Nuclear factor of activated T-cells, cytoplasmic, calcineurin-dependent 4, mRNA	*Nfatc4*	Forward	CCTGCCAGACTCTAAAGTGGT
			Reverse	TCAGTGTCACCTCACTGCTCTG
NM_017008.4	Glyceraldehyde-3-phosphate dehydrogenase, mRNA	*Gapdh*	Forward	GTGCCAGCCTCGTCTCATAG
			Reverse	AGAGAAGGCAGCCCTGGTAA
