# Supplementary material for: Dietary supplementation with bovine-derived milk fat globule membrane lipids promotes neuromuscular development in growing rats
Source: Nutr Metab (Lond). 2017 Jan 23;14:9. doi: 10.1186/s12986-017-0161-y (PMC5259894; doi:10.1186/s12986-017-0161-y)
Supplement: Additional file 2: Figure S1. — Real time RT‐PCR analysis of Agrn and Z+ Agrn expression in rat soleus muscle. Figure S2. Real time RT‐PCR analysis of synaptophysin expression in rat soleus muscle. A (PDF 369 kb) [file 12986_2017_161_MOESM2_ESM.pdf]

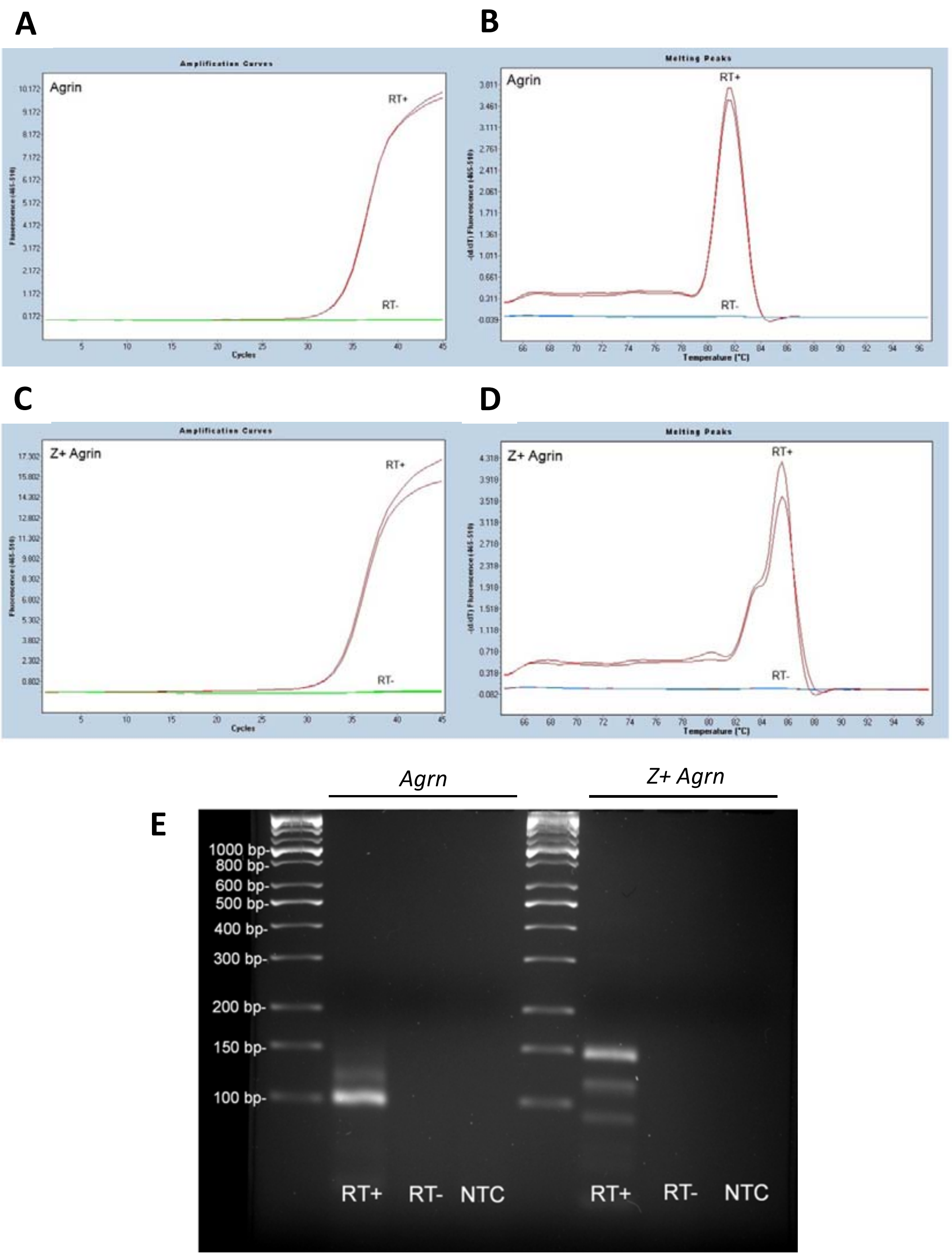

Supplemental Figure 1: Real time RT-PCR analysis of Agrn and Z+ Agrn expression in rat soleus muscle. A-D: Amplification plots and matching melting peaks for a pooled rat soleus muscle cDNA sample (RT+), the same RNA sample subjected to reverse transcription in the absence of reverse transcriptase enzyme (RT-), and a non-template control nuclear free water sample (NTC). E: Agarose gel (4%) electrophoresis of amplified product from the real time PCR reactions shown in A-D showing amplicon at expected size of 102 bp for *Agrn* (all variants) and 149 bp for nerve derived Z+ Agrn (Z8 and Z19 splice variants). Primers used for *Agrn Forward:* TTCCTCAGCAACTACAAACCTG, *Reverse:* TTCACACACAGCACCAAAGC. Primers used for Z+ *Agrn Forward:* TGTCCTGGGGGCTTCTCTGG and *Reverse* CTGGGATCTCATTGGTCAGCTC. ***Sequence of Agrn F1/Agrn R3 amplicon (102 bp band) as determined by gel extraction/purification and sequencing:***
TCAGCAACTACAAACCTGGAATCCACTTCACACCAGCGCCTCCTACGCCTCCTGATGTATGCAGGGGAATGTTATGTGGCTTTGGTGCTGTGTGT
GAA. ***Sequence of Agrn 30F/Agrn32R amplicon (149 bp band) as determined by gel extraction/purification and sequencing:***
GGGGCTTCTCTGGGCTGCACTGCGAAAAGGGGCTAGTTGAGAGTCAGTGGGGGACCTASAAACACTGGCCTTTGATGGGCSGAC.CTACATCR
AATACCTCAATGCTGTGATTGAGAGCGAGCTGACCAATGAGATCCCAG

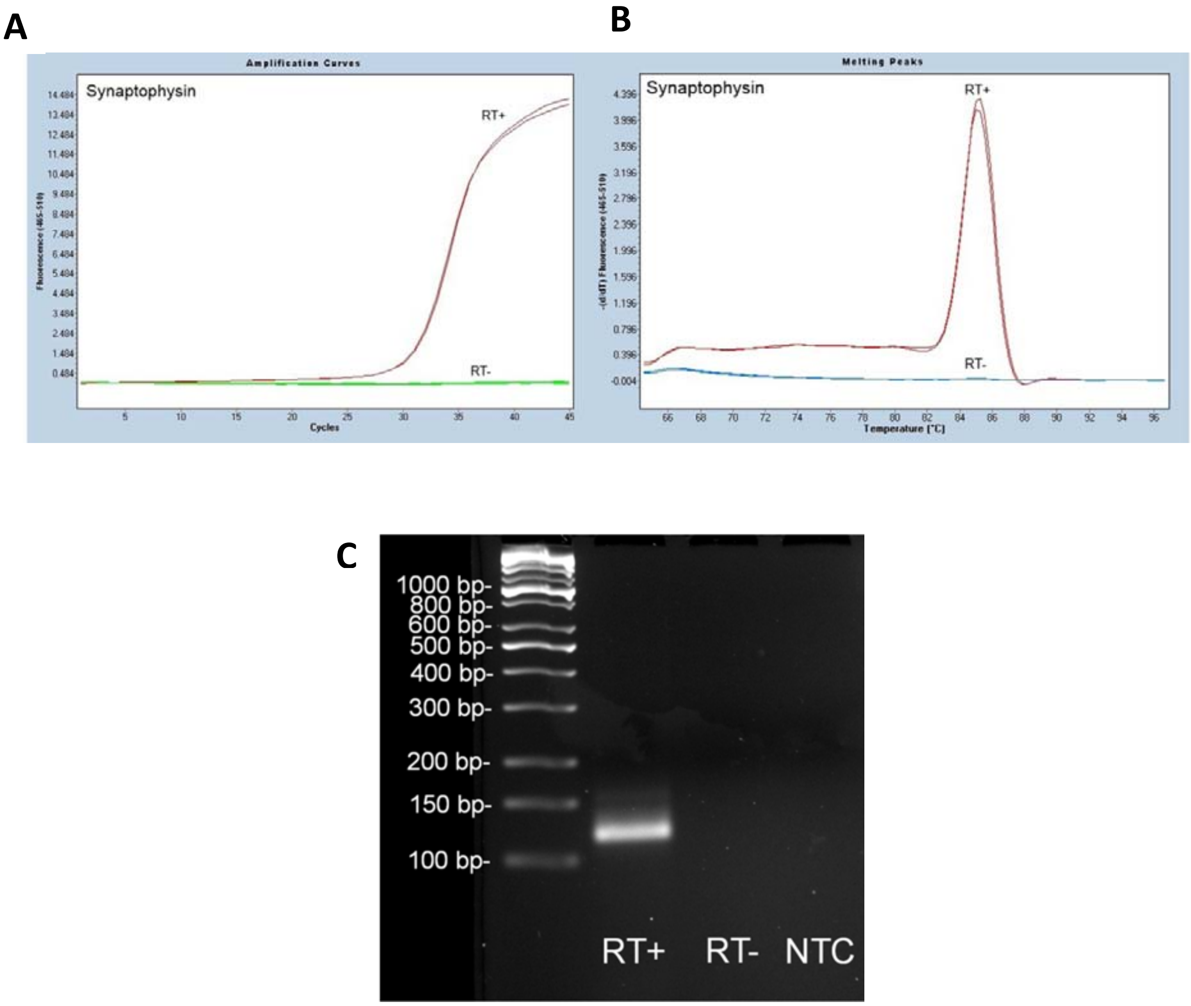

Supplemental Figure 2: Real time RT-PCR analysis of synaptophysin expression in rat soleus muscle. A: Amplification plots for a pooled rat soleus muscle cDNA sample (RT+), the same RNA sample subjected to reverse transcription in the absence of reverse transcriptase enzyme (RT-), and a non-template control (nuclear free water) sample (NTC). B: Matching real time RT-PCR melting peaks for pooled rat soleus muscle cDNA sample (RT+), the same RNA sample subjected to reverse transcription in the absence of reverse transcriptase enzyme (RT-), and a non-template control nuclear free water sample (NTC). C: Agarose gel (4%) electrophoresis of amplified product from the real time PCR reactions shown in A & B showing amplicon at expected size of 121 bp. Primers used *Forward:* TTTGCTACGTGTGGCAGCTA, *Reverse* ACACTTGGTGCAGCCTGAAG. ***Sequence of 121 bp band as determined by gel extraction/purification and sequencing:***
TTTTGCTACGTGTGGCAGCTACACCGGGGAGCTTCGGCTGAGCGTGGAGTGTGCCAACAAGACGGAGAGTGCCCTCAACAT
CGAAGTTGAATTCGAGTACCCCTTCAGGCTGCACCAGT
